# Transcriptome Analysis and Ultrastructure Observation Reveal that Hawthorn Fruit Softening Is due to Cellulose/Hemicellulose Degradation

**DOI:** 10.3389/fpls.2016.01524

**Published:** 2016-10-14

**Authors:** Jiayu Xu, Yuhui Zhao, Xiao Zhang, Lijie Zhang, Yali Hou, Wenxuan Dong

**Affiliations:** ^1^College of Horticulture, Shenyang Agricultural UniversityShenyang, China; ^2^College of Forestry, Shenyang Agricultural UniversityShenyang, China

**Keywords:** hawthorn, TEM observation, RNA-Seq, different texture, cell wall

## Abstract

Softening, a common phenomenon in many fruits, is a well coordinated and genetically determined process. However, the process of flesh softening during ripening has rarely been described in hawthorn. In this study, we found that ‘Ruanrou Shanlihong 3 Hao’ fruits became softer during ripening, whereas ‘Qiu JinXing’ fruits remained hard. At late developmental stages, the firmness of ‘Ruanrou Shanlihong 3 Hao’ fruits rapidly declined, and that of ‘Qiu JinXing’ fruits remained essentially unchanged. According to transmission electron microscopy, the middle lamella of ‘Qiu JinXing’ and ‘Ruanrou Shanlihong 3 Hao’ fruit flesh was largely degraded as the fruits matured. Microfilaments in ‘Qiu JinXing’ flesh were arranged close together and were deep in color, whereas those in ‘Ruanrou Shanlihong 3 Hao’ fruit flesh were arranged loosely, partially degraded and light in color. RNA-Seq analysis yielded approximately 46.72 Gb of clean data and 72,837 unigenes. Galactose metabolism and pentose and glucuronate interconversions are involved in cell wall metabolism, play an important role in hawthorn texture. We identified 85 unigenes related to the cell wall between hard- and soft-fleshed hawthorn fruits. Based on data analysis and real-time PCR, we suggest that β*-GAL* and *PE4* have important functions in early fruit softening. The genes *Ffase*, *Gns*,α*-GAL*, *PE63*, *XTH*, and CWP, which are involved in cell wall degradation, are responsible for the different textures of hawthorn fruits. Thus, we hypothesize that the different textures of ‘Qiu JinXing’ and ‘Ruanrou Shanlihong 3 Hao’ fruits at maturity mainly result from cellulose/hemicelluloses degradation rather than from lamella degradation. Overall, we propose that different types of hydrolytic enzymes in cells interact to degrade the cell wall, resulting in ultramicroscopic Structure changes in the cell wall and, consequently, fruit softening. These results provide fundamental insight regarding the mechanisms by which hawthorn fruits acquire different textures and also lay a solid foundation for further research.

## Introduction

Softening is a common phenomenon in many fruits and is an important factor that influences fruit quality. From horticultural and commercial viewpoints, ripening grants positive attributes to fruit and greatly impacts its various quality and nutritional characteristics, including fiber content and composition, lipid metabolism, and vitamin and antioxidant contents (Kalt, 2002). Ripening-related softening is a complex multi-component process. Fruit softening is attributed to the coordinated actions of several enzymes on different cell wall polymers. During the ripening process of fleshy fruits, it is generally accepted that textural changes, mainly associated with decreased firmness, result from the reduction of the cell to cell interactions due to the dissolution of the middle lamella, loosening of the primary cell wall and a reduction in cell turgor (Goulao and Oliveira, 2008; Mercado et al., 2011). During ripening, some fruits (e.g., kiwifruit, tomato, and European pear) soften to a melting texture, while other fruits (e.g., apple, watermelon, and Asian pear) partially soften and remain relatively firm and crisp (Ng et al., 2015). The extent of disassembly is mainly modified by pectin solubilization, neutral sugar loss, and xyloglucan depolymerization (Vicente et al., 2007; Sañudo-Barajas et al., 2009; Posé et al., 2013; Gwanpua et al., 2014), which result in fruit softening. These processes are thought to involve the coordinated and interdependent action of numerous enzymes that modify cell walls, such as polygalacturonase (*PG*), β-galactosidase (β*-GAL*), α-arabinofuranosidase (α*-ARF*), pectin methylesterase (*PME*), pectate lyase (*PL*), and xyloglucan endo-transglycosylase/hydrolase (*XTH*) (Brummell and Harpster, 2001; Giovannoni, 2001; Brummell, 2006; Goulao et al., 2008; Opazo et al., 2013). For example, in strawberry plants, the down-regulation of *PL* resulted in firmer fruits (Jiménez-Bermúdez et al., 2002). In one study, several transgenic plants with reduced cel1 mRNA levels were obtained, but no significant correlation was found with endoglucanase (*EG*) activity or fruit firmness (Woolley et al., 2001). Ortiz et al. (2010) indicated that a portion of the arabinose-rich side chains removed from the pectic polymers remained transiently linked to the chelator-soluble fraction of the cell wall. In addition, sugar analyses suggested that the disassembly of cell walls is aided by the previous elimination of galactan sidechains, which could facilitate pectin solubilization. The activity patterns of the considered cell wall-modifying enzymes were very similar. Given the complexity of the ripening process, the use of tools that allow global evaluations of the molecular processes triggered within the fruit are important. Some studies have already studied the softening of apple (Harb et al., 2012; Gwanpua et al., 2016), tomato (Lunn et al., 2013), peach (Yoshioka et al., 2011; Cao et al., 2014), strawberry (Posé et al., 2015), and pear (Hiwasa et al., 2004; Chiriboga et al., 2012; Wei et al., 2015) fruits.

The genus *Crataegus* (hawthorn), which is a common type of fruit, belongs to the Rosaceae family and is a genus of spiny trees or shrubs that exist in the northern hemisphere (Verma et al., 2007). Hawthorn that is planted for human consumption can be traced back to 300 A.D. In addition, hawthorn has been approved by the Ministry of Health of the People’s Republic of China as a raw material for functional foods and has been included in the Chinese Pharmacopeia as an herbal medicine (Liu et al., 2010). Furthermore, hawthorn is one of the most widely consumed horticultural products, either in fresh or processed form, and is an important component of many processed food products because of its excellent flavor, attractive color and high macro- and micro-nutrient contents (Cao et al., 1995; Özcan et al., 2005). To date, over 150 compounds, especially phenolic compounds, have been identified in *Crataegus pinnatifida* (Wu et al., 2014). Among these phenolic compounds, procyanidins (procyanidin B_2_, procyanidin B_5_, and procyanidin C_1_), flavonoids (epicatechin, hyperoside, quercetin, rutin, and isoquercitrin) and triterpenoids acid (ursolic acid, corosolic acid, oleanolic acid, and maslinic acid) are important bioactive components of hawthorn (Liu et al., 2011; Yang and Liu, 2012; Chai et al., 2014; Wu et al., 2014).

In recent years, Illumina RNA-Seq and digital gene expression (DGE) have provided an opportunity to explore different phenomena in diverse species via *de novo* assembly, facilitating the identification and analysis of the majority of transcriptomes (Zhang et al., 2010). Using RNA-Seq technology, Dai et al. (2013) found that degraded fruit endocarp (stone) formation might be correlated with decreased lignin synthesis in a unique hawthorn variety, ‘soft-endocarp,’ which has a soft and broken endocarp (stone), whereas the endocarp of most hawthorn varieties are hard. Nevertheless, few data are available regarding the different textures of hard- and soft-fleshed hawthorn fruits. Almost 300 resources are present in the National Hawthorn Germplasm Repository of Shenyang, China. ‘Ruanrou Shanlihong 3 Hao’ is a wild cultivar of *C. pinnatifida* with lower flesh firmness during the fruit maturing and harvesting periods than hawthorn fruits with average firmness. Thus, ‘Ruanrou Shanlihong 3 Hao’ fruit is a preferable fruit for studying fruit softening. ‘Qiu JinXing’ is an existing cultivar with fruits that are firmer during fruit ripening and with good fruit qualities and properties. Therefore, ‘Qiu JinXing’ and ‘Ruanrou Shanlihong 3 Hao’ were chosen as experimental materials for this study. Transmission electron microscopy (TEM) was used to observe the microstructure of the fruit flesh cells and Illumina-based RNA-Seq was used to elucidate differential gene expression (Shin et al., 2015). For this analysis, ultrathin sections of hawthorn flesh were obtained and a *de novo* assembly of the fruit transcriptome of *C. pinnatifida* was performed. Using RNA-Seq, the hawthorn transcriptomes were sequenced, assembled, and annotated for the hawthorn fruits with hard and soft flesh. Using these platforms, the global transcriptional analysis of gene expression in the hawthorn fruits with hard and soft flesh were described. When determining the different textures of the hawthorn fruit cell walls, the cell wall metabolism was investigated at the molecular level in addition to the relationships between the important enzymes in the cell wall. Fruit softening is of theoretical significance and should be quantified to further reveal the mechanisms of hawthorn fruit softening during maturation.

## Materials and Methods

### Plant Material

**FIGURE 1:**
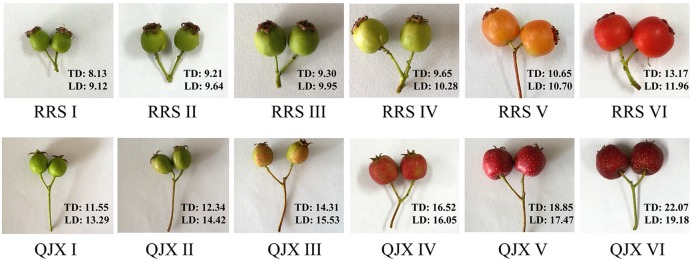
**Different stages of ‘Ruanrou Shanlihong 3 Hao’ and ‘Qiu JinXing’ fruit development.** TD, transverse diameter (mm); LD, longitudinal diameter (mm).

The hard-fleshed hawthorn ‘Qiu JinXing’ and the soft-fleshed hawthorn ‘Ruanrou Shanlihong 3 Hao’ were maintained in the National Hawthorn Germplasm Repository of China at Shenyang. Fruits were collected at the early developmental stageI(RRSI- 90 days before harvesting ‘Ruanrou Shanlihong 3 Hao’; QJXI- 105 days before harvesting ‘Qiu JinXing’), early developmental stage IIII (RRSII- 75 days before harvesting ‘Ruanrou Shanlihong 3 Hao’; QJXII- 75 days before harvesting ‘Qiu JinXing’), middle developmental stageI(RRSIII- 60 days before harvesting ‘Ruanrou Shanlihong 3 Hao’; QJXIII- 45 days before harvesting ‘Qiu JinXing’), middle developmental stageII(RRSIV- 45 days before harvesting ‘Ruanrou Shanlihong 3 Hao’; QJXIV- 30 days before harvesting ‘Qiu JinXing’), late developmental stageI (RRSV- 15 days before harvesting ‘Ruanrou Shanlihong 3 Hao’; QJXV- 15 days before harvesting ‘Qiu JinXing’), and late developmental stageII (RRSVI- the day of harvest of ‘Ruanrou Shanlihong 3 Hao’; QJXVI- the day of harvest of ‘Qiu JinXing’) (**Figure 1**). Fruits were selected for their uniform size, appearance, and lack of defects. Fifteen fruits were analyzed to determine their firmness and weight at different developmental stages. The remaining fruits were frozen in liquid N_2_ and stored at -80°C for further analysis.

### Measurement of Fruit Firmness and Weight of Hawthorn Fruits with Hard and Soft Flesh

Fruit firmness was measured on the opposite sides of each individual fruit among the 15 analyzed fruits at different developmental stages by using a non-damage texture analyzer (test anvil, cylinder 0.10 cm^2^; spring force, 806.50 mN; contact pressure, 12.50 N; resolution, 0.10 Fff; HPE II Fff, Bareiss, Germany), and the maximum force developed during the test was recorded. Firmness was determined as the average force in Newtons (N). Fruit total sugar, titrable acidity were measured using the method according to Ranganna (2001). An electronic analytical balance (YP1002N, Shanghai jingke, China) was used to measure the fruit weight at different developmental stages, and all statistical analyses were conducted using SPSS 19.0.

### Observations of the Ultrastructures of the Fruit Cell Walls

The flesh tissues of the selected ‘Ruanrou Shanlihong 3 Hao’ and ‘Qiu JinXing’ fruits sampled at different developmental stages were fixed in 2.5% glutaraldehyde and 1% osmic acid, washed with PB buffer solution, dewatered using a gradient of ethanol concentrations, embedded with SPI812 resin, sliced using an Leica-EM-UC 7 ultramicrotome (thickness of 90 nm; Germany), dyed with uranyl acetate and lead citrate, and observed and photographed using a HT – 7700 TEM (Hitachi, Japan).

### RNA Isolation, cDNA Library Preparation and Sequencing

Total RNA isolated from flesh was extracted using the modified CTAB method according to Chang et al. (2007). The RNA purity was assessed using a NanoPhotometerR spectrophotometer (IMPLEN, USA), and the RNA concentration was measured using Qubit^®^ RNA Assay Kit (Q32852) with a Qubit2^®^.0 Fluorometer (Q32857; Life Technologies, USA). The quality of total RNA was evaluated using an Agilent Bioanalyzer 2100 system (G2939AA, Agilent Technologies, USA). Only samples with an RNA integrity number (RIN) ≥7 and a 28S:18S RNA ratio ≥1.5 were used for deep sequencing. For mRNA library construction and deep sequencing, RNA samples were prepared using NEBNext^®^Ultra^TM^ RNA Library Prep Kit for Illumina^®^ (E7530, NEB, USA) according to the manufacturer’s protocol, including polyA-mRNA purification and fragment, first-strand cDNA synthesis, second-strand cDNA synthesis and repair, adapter ligation, PCR enrichment, agarose gel purification, and library quality control. cDNA samples were prepared from the fruits of hard- and soft-fleshed hawthorns and sequenced using Illumina HiSeq^®^ 2500. cDNA library preparation and sequencing reactions were conducted by the Biomarker Technology Company, Beijing, China. Paired-end library preparation and sequencing were performed following standard Illumina methods.

### *De novo* Transcriptome Filtering, Assembly and Annotation

Four samples, including samples of the two hard-fleshed hawthorns QJXIV (‘Qiu JinXing’ fruit at the middle developmental stageII) and QJXV (‘Qiu JinXing’ fruit at the late developmental stage I), the two soft-fleshed hawthorns RRSIV (‘Ruanrou Shanlihong 3 Hao’ fruit at the middle developmental stageII) and RRSV (‘Ruanrou Shanlihong 3 Hao’ fruit at the late developmental stageI), were sequenced. Each of the samples had two biology repeats. Raw data (raw reads) in the fastq format were filtered by removing the reads containing the adaptor, including the reads whose poly-*N* content was more than 10% and the reads with more than 50% bases and whose quality score Q was no more than five from the raw data. The remaining data were termed clean data (clean reads). Simultaneously, the Q20, Q30, GC-content and sequence duplication levels of the clean data were calculated. The following analyses were based on clean data. The left file was named left.fq, and the right file was named right.fq. Transcriptome assembly was accomplished based on left.fq and right.fq using Trinity (Grabherr et al., 2011) with min-kmer-cov set to two and all other parameters set to default values. Based on the NR (Deng et al., 2006), Swiss-Prot (Apweiler et al., 2004), gene ontology (GO) (Ashburner et al., 2000), COG (Tatusov et al., 2000), KOG (Koonin et al., 2004), and KEGG (Kanehisa et al., 2004) databases, BLAST (Altschul et al., 1997) was used to achieve the amino acid sequences of the unigenes and the HMMER (Finn et al., 2013) software and Pfam (Eddy, 1998) database alignment were used to obtain annotated unigene information. The raw data has been deposited at NCBI database, and the accession number of SRA database is: SRP082543.

### Differential Expression Analysis

Gene expression levels were measured in the RNA-Seq analysis as reads per kilobase of exon model per million mapped reads FPKM (Trapnell et al., 2010). The DESeq software (Anders and Huber, 2010) was used to identify differentially expressed genes in a pairwise comparison, and the false discovery rate (FDR) and fold change (FC) were used to determine the DEGs. In our study, genes with FDR ≤ 0.01 and FC ≥ 2 in FPKM were considered as DEGs.

### Quantitative Reverse Transcriptase Real-time PCR Analysis

First-strand cDNA was synthesized from 1 μg of RNA using PrimeScriptTM RT Reagent Kit with gDNA Eraser (RR047A, TaKaRa, China) following the manufacturer’s protocol. The synthesized cDNA was diluted 10-fold for the following Real-Time PCR analysis. Primers for all selected genes were designed using Primer Prime 5 software. The annealing temperature and primer sequence of the analyzed amplicons are listed in **Supplementary Table S2**, and amplification products were sequenced (**Supplementary Figure S1**) to check the product identity. For each gene, a cDNA serial dilution was used to generate a standard curve to calculate efficiencies. Quantitative PCR was performed using an iQ5 Real-Time PCR detection system (Bio-Rad, USA) with 20 μl reactions including 1 μl diluted cDNA sample, 1 μl each primer (10 μM) and 10 μl SYBR Green Master Mix (RR820A, Takara, China) under the following conditions: an initial hot start at 95°C for 3 min, followed by 40 cycles of 95°C for 10 s, the annealing temperature for each selected gene for 30 s and 72°C for 30 s, following melting curve range from 50 to 90°C. Two internal control genes, *Actin* and *NADPH*, were used for normalization, and relative gene expression was estimated using threshold cycles and the 2^-ΔΔCT^ method (Livak and Schmittgen, 2001). Real-time PCR analysis was carried out with three biological and three technical replicates.

## Results

### Changes of Fruit Firmness, Weight, Total Sugar, and Titratable Acidity at Different Fruit Developmental Stages

Fruit firmness is an important factor that affects fruit texture, ripening and softening. At the early and middle stages of fruit developmental, the firmness of ‘Ruanrou Shanlihong 3 Hao’ and ‘Qiu Jingxing’ fruits were maintained at a high level, with the value of firmness of the two cultivars almost equal. However, at developmental stages V and VI, the firmness of ‘Ruanrou Shanlihong 3 Hao’ fruits decreased rapidly, whereas that of ‘Qiu JinXing’ declined slowly and remained at a significantly higher level (**Table 1**). At maturity, the firmness of ‘Qiu JinXing’ fruits was higher than that of ‘Ruanrou Shanlihong 3 Hao’ fruits (**Table 1**). With fruit development, the fruit weights of ‘Ruanrou Shanlihong 3 Hao’ (soft flesh) and ‘Qiu Jingxing’ (hard flesh) gradually increased, accumulation of total sugar in the two cultivars were declined at early developmental stageII, and then increased gradually (**Table 1**). Titratable acidity contents of ‘Ruanrou Shanlihong 3 Hao’ fruit were increased gradually and reached to a peak value at middle developmental stage I and then decreased, whereas that in ‘Qiu Jinxing’ fruit were increased continuously (**Table 1**).

**Table 1:** Variations in fruit firmness, weight, total sugar and titratable acidity in ‘Ruanrou Shanlihong 3 Hao’ and ‘Qiu JinXing’ fruits.

Cultivar	Developmental stage	Fruit firmness (*N*)	Fruit weight (*g*)	Total sugar (%)	Titratable acidity (%)
Ruanrou Shanlihong 3 Hao	I	44.64 ± 0.19b	0.32 ± 0.01j	0.99 ± 0.01ih	0.62 ± 0.01e
	II	44.98 ± 0.33b	0.47 ± 0.01ij	0.81 ± 0.02ih	0.66 ± 0.00e
	III	45.66 ± 0.35b	0.52 ± 0.01ij	1.00 ± 0.03h	0.99 ± 0.02c
	IV	45.38 ± 0.16b	0.57 ± 0.01hi	1.42 ± 0.08g	0.97 ± 0.01c
	V	39.54 ± 0.41c	0.75 ± 0.02gh	2.23 ± 0.06e	0.85 ± 0.01d
	VI	28.89 ± 0.58d	1.16 ± 0.02ef	2.90 ± 0.03c	0.82 ± 0.01d
Qiu JinXing	I	44.26 ± 0.29b	0.94 ± 0.02fg	1.53 ± 0.06g	0.66 ± 0.01e
	II	47.97 ± 0.20a	1.27 ± 0.01e	0.70 ± 0.03i	0.88 ± 0.01d
	III	46.37 ± 0.19ab	1.91 ± 0.03d	1.80 ± 0.08f	1.02 ± 0.01c
	IV	46.11 ± 0.33ab	2.67 ± 0.03c	2.54 ± 0.17d	1.22 ± 0.02b
	V	41.30 ± 0.65c	3.52 ± 0.05b	5.27 ± 0.16b	2.94 ± 0.04a
	VI	40.03 ± 0.21c	4.93 ± 0.09a	7.25 ± 0.12a	2.99 ± 0.06a

*I, early developmental stage I; II, early developmental stage II; III, middle developmental stage I; IV, middle developmental stage II; V, late developmental stage I; and VI, late developmental stage II. Different letters indicate significant differences among the treatments at p < 0.05*.

### Ultramicroscopic Structure of the Fruit Flesh at Different Fruit Developmental Stages

**FIGURE 2:**
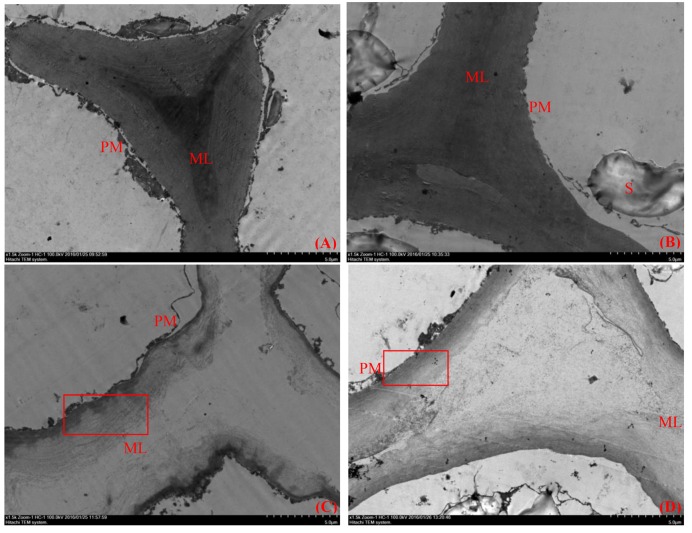
**Changes in the ultramicroscopic structure of cell walls in hawthorn fruits at different fruit developmental stages. (A)** The middle developmental stage of ‘Ruanrou Shanlihong 3 Hao’ fruits; **(B)** the middle developmental stage of ‘Qiu JinXing’ fruits; **(C)** the late developmental stage of ‘Ruanrou Shanlihong 3 Hao’ fruits; **(D)** the late developmental stage of ‘Qiu JinXing’ fruits. ML, middle lamella; PM, cell membrane; S, starch grain.

Plant cell wall structure is an important factor that determines fruit firmness, and plant cell walls are complex structures composed of cellulose, hemicellulose, pectin and protein. TEM can be used to observe the ultramicroscopic structure of fruit flesh tissue clearly. At the middle developmental stages of ‘Ruanrou Shanlihong 3 Hao’ and ‘Qiu JinXing,’ the structures of the cell walls in the fruit flesh were complete, presenting a light-dark-light structure and a plasma membrane located next to the cell wall. Moreover, deep-colored and closely arranged microfilaments and dark middle lamella were observed (**Figures 2A,B**). At the late developmental stage, the middle lamella of ‘Ruanrou Shanlihong 3 Hao’ and ‘Qiu JinXing’ fruit flesh tissue was nearly all degraded, the light-dark-light structure disappeared, and plasmolysis occurred (**Figures 2C,D**). However, the microfilaments in the ‘Qiu JinXing’ fruit tissue were arranged closely and colored deeply (**Figure 2D**), whereas the microfilaments in the ‘Ruanrou Shanlihong 3 Hao’ fruit flesh tissue were arranged loosely, partially degraded and became lighter in color (**Figure 2C**). The trend of the changes in fruit firmness was nearly consistent with the TEM observations.

### RNA-Seq and *De novo* Transcriptome Assembly

In the RNA-Seq experiment of ‘Ruanrou Shanlihong 3 Hao’ and ‘Qiu JinXing’ fruits from two different developmental stages, approximately 46.72 Gb of clean data were obtained for all the samples in the RNA-Seq. In addition, 2,182,914 contigs, 199,204 transcripts, and 72,837 unigenes were obtained, and the N50s of the contigs, transcript and unigene were 88, 1,656, and 1,283, which indicated a high assembly integrity. The total lengths of the contigs, transcript and unigene were 168,024,365, 210,439,324, and 50,660,379, respectively. The mean lengths of the contigs, transcript and unigene were 76.97, 1056.40, and 695.53, respectively. Among the unigenes, 29,081 unigenes (39.93%) had lengths of 200–300 nt, 17,945 unigenes (24.64%) had lengths ranging from 300 to 500 nt, 11,447 unigenes (15.72%) had lengths ranging from 500 to 1,000 nt, 8,837 unigenes (12.13%) had lengths ranging from 1,000 to 2,000 nt, and 5,527 unigenes (7.59%) had lengths of more than 2,000 nt, resulting in a mean length of 695.53 (**Table 2**).

**Table 2:** The length distribution of assembled unigenes.

Length range	Contig	Transcript	Unigene
200-300	2,125,350 (97.36%)^∗^	39,934 (20.05%)	29,081 (39.93%)
300-500	26,794 (1.23%)	34,334 (17.24%)	17,945 (24.64%)
500-1000	15,601 (0.71%)	43,252 (21.71%)	11,447 (15.72%)
1000-2000	9,646 (0.44%)	53,297 (26.75%)	8,837 (12.13%)
2000+	5,523 (0.25%)	28,387 (14.25%)	5,527 (7.59%)
Total number	2,182,914	199,204	72,837
Total length	168,024,365	210,439,324	50,660,379
N50 length	88	1,656	1,283
Mean length	76.97	1056.40	695.53

*The values in the tables show the numbers of contig/transcript/unigene in the corresponding range. The percentages in the brackets show the ratio of contig/transcript/unigene in the corresponding range. The table of ^∗^ shows the numbers and percentages of contig with lengths of 0–300.*

### Functional Annotation and Classification

Transcriptome analysis is important for elucidating the molecular components of cells and tissues and for interpreting the functional elements of the genome (Qiu et al., 2013). To classify the potential functions of the assembled unigenes, a sequence similarity search was conducted against the NR, Swiss-Prot, GO, COG, KOG, KEGG, and Pfam databases. A threshold magnitude of less than 10^-5^ was used for the NR, Swiss-Prot, GO, COG, KOG, KEGG databases and a threshold magnitude of less than 10^-10^ was used for the Pfam database. The results showed that 12,143, 27,411, 8,280, 20,603, 22,628, 22,240, and 39,248 unigenes exhibited significant similarity to known protein genes in the seven databases. Overall, 39,701 unigenes were annotated in the seven databases, including 28,466 unigenes with lengths of more than 300 nt, and 13,682 unigenes with length of more than 1000 nt (**Table 3**).

**Table 3:** Summary of unigene annotations.

Annotated databases	Unigene	≥300 nt	≥1000 nt
COG	12,143	9,496	57,71
GO	27,411	20,515	11,163
KEGG	8,280	6,293	3,305
KOG	20,603	15,689	8,557
Pfam	22,628	18,704	11,477
Swiss-Prot	22,240	17,996	10,150
nr	39,248	28,287	13,661
All	39,701	28,466	13,682

*Annotated database, different functional databases; Unigene, unigene value of annotated databases; ≥300 nt, the unigene value of annotated databases with a length of more than 300 bases; ≥1000 nt, unigene value of annotated databases with a length of more than 1000 bases.*

Based on the nr annotation, 19,887 of the distinct sequences showed top matches with sequences from *Prunus persica*, 3,393 of the distinct sequences showed top matches with sequences from *Fragaria vesca*, and only 737 and 704 of the distinct unigene sequences showed top matches with sequences from *Bipolaris maydis* and *Theobroma cacao*, respectively (**Supplementary Figure S2**).

**FIGURE 3:**
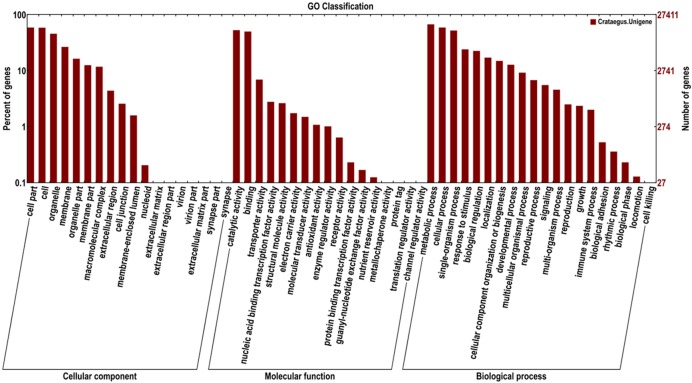
**Histogram of the GO classifications of the assembled *C. pinnatifida* unigenes.** Most consensus sequences were grouped into three major functional categories, namely, biological processes, cellular components, and molecular functions.

Based on the GO databases, 27,411 putative unigenes of *C. pinnatifida* were categorized into 55 functional groups that belonged to three main GO ontologies: cellular component, molecular function, and biological process (**Figure 3**). Next, 71,981, 33,536, and 43,271 unigenes were annotated as the biological process, cellular component and molecular function, respectively. The ‘Cell part,’ ‘Cell,’ ‘catalytic activity,’ and ‘metabolic process’ terms were most prevalent. In addition, a high percentage of genes were classified under the ‘Binding’ and ‘Cellular process’ terms, and only a few genes were classified as ‘protein tag,’ ‘Virion,’ and ‘synapse part’ (**Figure 3**).

Based on the KOG databases, 12,143 putative unigenes of *C. pinnatifida* were categorized into 25 functional groups. The cluster for ‘General function prediction only’ was larger than any other group. However, few genes of the ‘cell motility’ category were found (**Supplementary Figure S3**).

The KEGG pathway is a collection of manually drawn pathway maps on the molecular interaction and reaction network. Pathway analysis provides information regarding biological functions and gene interactions. When all the annotated unigenes were searched for against the KEGG pathway, 8,280 unigenes were mapped to 120 pathways. Among the 120 KEGG pathways, galactose metabolism and pentose and glucuronate interconversions occurred in the cell wall, which suggested that the these pathways may play a role in different hawthorn textures.

### DEG Expression

**FIGURE 4:**
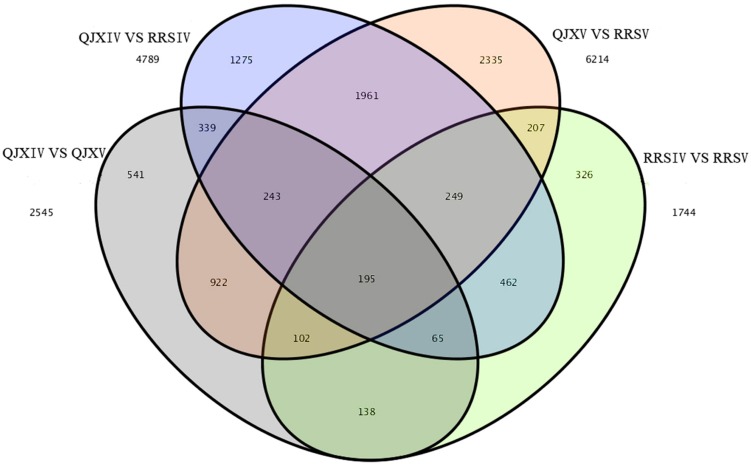
**Venn map showing the unique and shared unigenes with significantly different expression levels in ‘Ruanrou Shanlihong 3 Hao’ and ‘Qiu JinXing’ fruits at two developmental stages**.

Among the four samples, 195 common and different unigenes were found, with 6214 and 4789 different unigenes in RRSV and QJXV and in RRSIV and QJXIV, respectively (**Figure 4**). Some genes were down-regulated in RRSIV and QJXIV but obviously up-regulated in RRSV and QJXV, and some genes showed increased quantitative expression of RRSIV and RRSV but decreased transcript abundance in QJXIV and RRSVI. We identified 85 distinctly different unigenes with functions related to cell walls among the hawthorns with hard and soft flesh. Galactose metabolism and pentose and glucuronate interconversions were related to fruit conversion, with different degrees of softening in hawthorn fruits with soft and hard flesh.

### Genes Related to the Different Texture of Hard- and Soft-Fleshed Hawthorn during Two Periods

**FIGURE 5:**
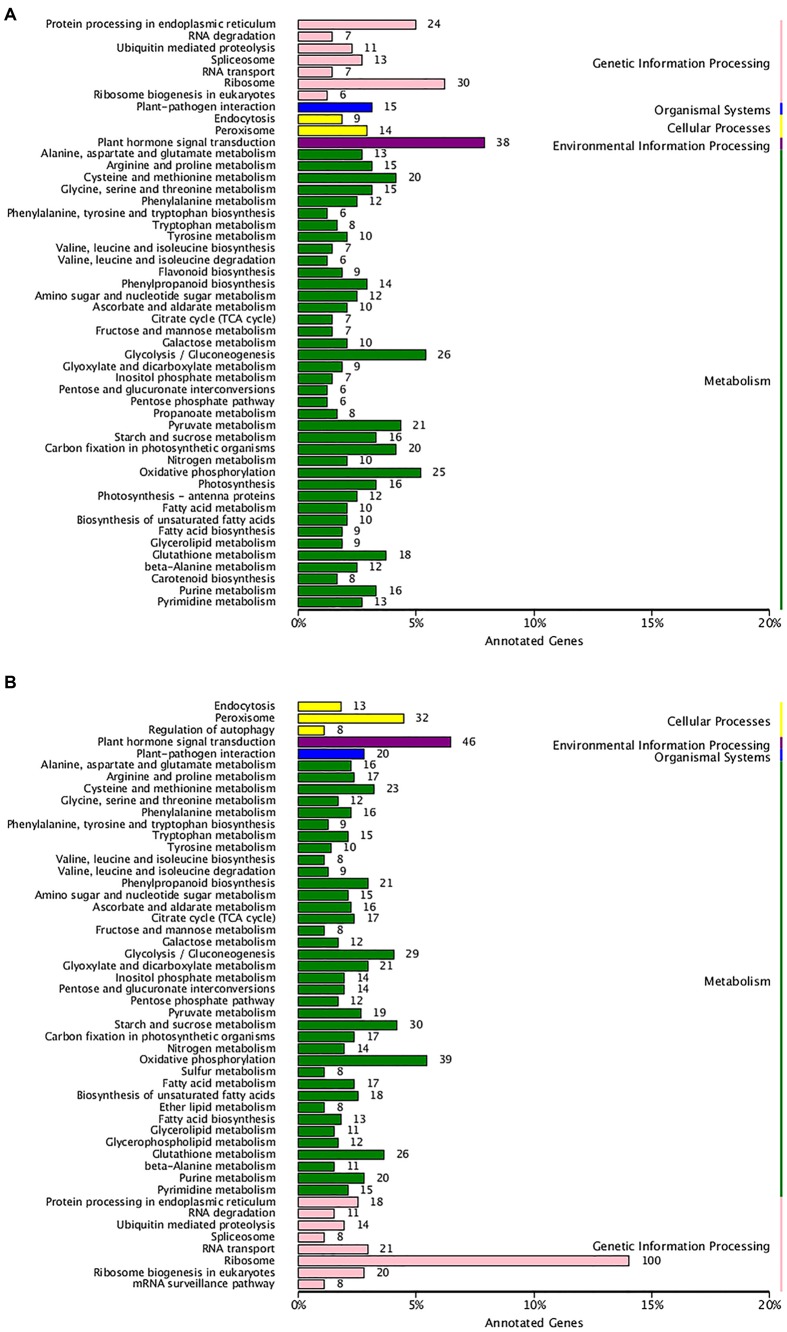
**The enriched pathways of significant DEGs. (A)** For RRSIV and QJXIV and **(B)** for RRSV and QJXV. Ordinate: nomenclature of the KEGG metabolic pathway; abscissa: gene numbers that annotated this pathway and the ratio of KEGG in all genes that annotated relative to the total KEGG.

To examine the biochemical basis of different textures between two types of hawthorns during the two periods, we performed transcriptome analysis. Expression analysis was conducted for two pairwise comparisons: RRSIV vs. QJXIV and RRSV vs. QJXV. Overall, 2377 up-regulated and 2412 down-regulated unigenes were observed between RRSIV and QJXIV (**Supplementary Figure S4A**), and 3434 up-regulated and 2780 down-regulated unigenes were observed between RRSVand QJXV (**Supplementary Figure S4B**). To appreciate the biological importance of the 4,789 differentially expressed genes between RRSIV and QJXIV, the lists of significantly down-regulated or up-regulated genes were analyzed with the database for annotation, and 43 unigenes (**Supplementary Table S1**) with functions related to cell wall structure were expressed differently. Overall, 10 different unigenes were used in galactose metabolism, 6 unigenes were used in pentose and glucuronate interconversions, and so on (**Figure 5A**). More differences were observed in the chloroplast (GO: 0009507), and the second plasma membrane (GO: 0005886) (**Supplementary Figure S5A**). In the list of 19 up-regulated genes (**Supplementary Table S1**), the terms of ‘*Ffase*’ and ‘β*-GAL*’ were over-represented. To appreciate the biological importance of the 6,789 differentially expressed genes between RRSV and QJXV, the lists of significantly down-regulated or up-regulated genes were analyzed using the database for annotation, and 66 unigenes (**Supplementary Table S1**) with functions related to the cell wall were expressed differently. Overall, 12 different unigenes were expressed in galactose metabolism, 14 unigenes were expressed in pentose and glucuronate interconversions and so on (**Figure 5B**). More unigenes were found to be expressed in chloroplasts (GO: 0009507) and the plasma membrane (G: 0005886) (**Supplementary Figure S5B**). In the list of 45 up-regulated genes, ‘*Gns*’ and ‘*Ffase*’ are the most over-represented genes in terms of the different expressed genes. The expression of CPW in ‘Qiu JinXing’ fruit was higher than that in ‘Ruanrou Shanlihong 3 Hao’ fruit in both RRSIV vs. QJXIV and RRSV vs. QJXV. The expression of glycine-rich structural proteins and thin1 proteins in the cell walls in ‘Ruanrou Shanlihong 3 Hao’ fruit was higher than in ‘Qiu JinXing’ fruit in both RRSIV vs. QJXIV and RRSV vs. QJXV.

### Verification of Gene Expression by Real-time PCR

**FIGURE 6:**
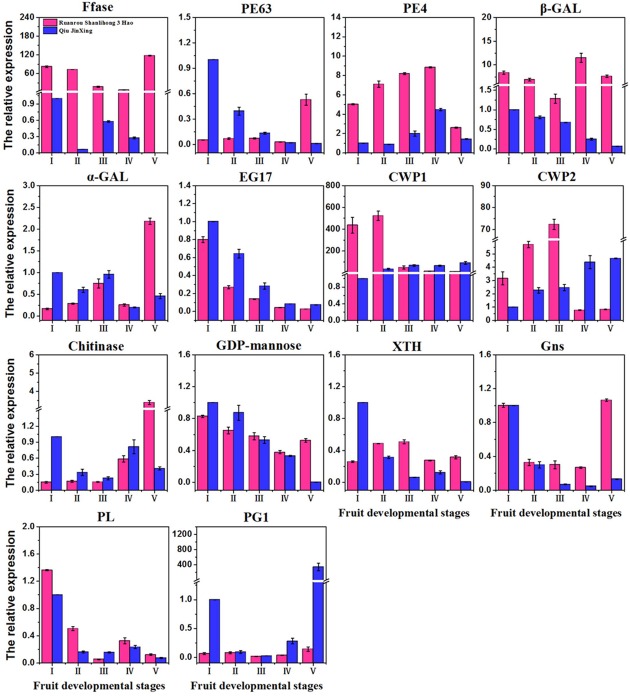
**Quantitative real-time PCR validation of 14 DEGs of ‘Ruanrou Shanlihong 3 Hao’ and ‘Qiu JinXing’ fruits.** *Ffase*, β-fructofuranosidase; *PE*, pectinesterase; β*-GAL*, β-galactosidase; α*-GAL*, α-galactosidase; *EG*, endoglucanase; CWP, cell wall protein; *GDP-mannose*, glucomannan 4-β-mannosyltransferase; *XTH*, xyloglucan glycosyltransferase; *Gns*, glucan endo-1,3-β-D-glucosidase; *PL*, pectate lyase; and *PG*, polygalacturonase.

To confirm the differential expression profiles of DEGs identified from RNA-Seq analysis, we selected 14 candidate DEGs with high expression levels for real-time assays. The expression levels of 14 DEGs at different fruit developmental stages were assayed. Real-time PCR indicated that the expression patterns of the 14 DEGs fitted those determined by RNA-Seq analysis. For instance, several up-regulated DEGs (β*-GAL*) with highly abundant transcripts also exhibited relatively high levels of expression in real-time PCR analysis (**Figure 6**). The FCs of some DEGs from real-time PCR were different from those obtained from RNA-Seq analysis, partially due to the different sensitivities of the two approaches and their different algorithms. The RNA-Seq generated absolute expression patterns, and real-time PCR showed relative expression values at the transcriptional level.

## Discussion

Cell wall degradation is the main factor affecting fruit softening and quality (Bennett and Labavitch, 2008). With the progression of ripening, complex carbohydrates are converted to simple sugars, and the acidity decreases with the accumulation of sugar. Meanwhile, as aromatic compounds accumulate, the cell wall dynamics change, which results in either dehiscence or softening (Klee and Giovannoni, 2011). In previous studies, degradation of the cell wall network structure was observed during the process of fruit maturation in tomato (Orfila et al., 2002). In addition, at the late stage of pineapple softening, the electron density of the middle lamella decreased, the cell wall became uneven, and microfibrils in the cell wall became loose and disordered (Khalil et al., 2007). In this study, the process of fruit softening during the maturation of ‘Ruanrou Shanlihong 3 Hao’ and ‘Qiu JinXing’ fruits was accompanied by fruit cell wall structural changes. The middle lamella of the ‘Ruanrou Shanlihong 3 Hao’ and ‘Qiu JinXing’ fruit flesh tissue were nearly completely degraded. However, the degree of microfilament degradation in the ‘Ruanrou Shanlihong 3 Hao’ fruit flesh was greater than that in ‘Qiu JinXing’ fruit flesh. These results indicated that the difference in fruit firmness between ‘Ruanrou Shanlihong 3 Hao’ and ‘Qiu JinXing’ fruits at maturity mainly resulted from cellulose/hemicellulose degradation rather than middle lamella degradation.

We obtained 46.72 Gb of clean data by RNA-Seq, with 2,182,914 contigs, 199,204 transcripts, and 72,837 unigenes in RNA-Seq. Overall, 12,143, 27,411, 8,280, 20,603, 22,628, 22,240, and 39,248 unigenes exhibited significant similarity to the known protein genes in the NR, Swiss-Prot, GO, COG, KOG, KEGG, and Pfam databases, respectively, and some KEGG pathways. With the application of massive and parallel sequencing technologies, transcriptome information has become generally abundant for not only model organisms on which international research efforts and funding are concentrated but also for non-model organisms. These results add to the current data on *C. pinnatifida* and provide data for future research in this species. In this study, RNA-Seq technology transcriptome analysis of hard- and soft-fleshed hawthorn revealed that 85 hydrolytic enzyme genes (**Supplementary Table S1**) and two different signaling pathways, galactose metabolism and pentose and glucuronate interconversions, related to cell wall processes are associated with cell wall degradation.

In galactose metabolism, β*-GAL* was usually proposed to modify the cell wall via the removal of galactosyl and arabinosyl residues from polysaccharides in the cell wall at the initiation of fruit ripening (Mwaniki et al., 2007; Martínez and Civello, 2008; Chávez Montes et al., 2008; Posé et al., 2013). High β*-GAL* activity is very important during early ripening of Jonagold apples (Gwanpua et al., 2014), which is supported by our results. In our study, at the middle developmental stageII, a time when hawthorn fruits begin to soften, the expression levels of β*-GAL* were higher in ‘Ruanrou Shanlihong 3 Hao’ fruits than that in ‘Qiu JinXing’ fruits. Furthermore, α*-GAL* and *Ffase*, which are very important to such metabolism, were found for the first time in this study to be related to fruit softening. Before fruit firmness decreased, the level of α*-GAL* gene expression in ‘Qiu JinXing’ fruits was higher than that in ‘Ruanrou Shanlihong 3 Hao’ fruits. When the hawthorn fruits were softening, the level of α*-GAL* expression in ‘Ruanrou Shanlihong 3 Hao’ increased abruptly, though a continuous low level of expression was observed in ‘Qiu JinXing’ fruits, with higher levels of gene expression in ‘Ruanrou Shanlihong 3 Hao’ fruits compared to ‘Qiu JinXing’ fruits. The result suggested that α*-GAL* plays a role in changing the fruit texture. During the process of ‘Qiu JinXing’ fruit development in this experiment, the *Ffase* expression level was weak while the expression level of *Ffase* was high in the soft-fleshed hawthorn during the process of fruit development, which indicated that *Ffase* had an important impact on the fruit texture. In pentose and glucuronate interconversions, the level of *PE63* expression generally decreased with increasing fruit development in ‘Qiu JinXing’ fruits. In contrast, in ‘Ruanrou Shanlihong 3 Hao’ fruits, the gene expression level was weak at the early and middle developmental stages and rapidly increased at the late developmental stage. The expression levels of *PE63* were higher in ‘Ruanrou Shanlihong 3 Hao’ fruits at the late developmental stage, a time when hawthorn fruits were softening, showing that *PE63* plays a role in hawthorn fruit softening. The *PE4* expression level showed an increasing then decreasing tendency across the fruit developmental stages in the two cultivars. At middle developmental stageII, when hawthorn fruits begin to soften, the expression levels of *PE4* were higher in ‘Ruanrou Shanlihong 3 Hao’ fruits than in ‘Qiu JinXing’ fruits. Posé et al. (2015) observed that silencing of *PL* genes reduced pectin degradation during strawberry fruit softening, though this is inconsistent with our results. In our study, the *PL* expression showed a decreasing, increasing and decreasing tendency at the early, middle and late fruit developmental stages, respectively, and *PL* expression in the two cultivars did not differ during fruit softening. Therefore, we hypothesize that *PL* does not affect hawthorn fruit softening. Overall, these results demonstrate *PE4 and* β*-GAL* has an important function in the early softening of different textures; α*-GAL*, *Ffase*, and *PE63* might play key roles in textural changes, though the crucial participation of α*-GAL* and *Ffase* in fruit softening is reported for the first time in this study.

Costa et al. (2010) used QTL analysis to show that *PG1* expression was associated with fruit firmness in apples, which was later validated by Longhi et al. (2013). Furthermore, Atkinson et al. (2012) showed that *PG*-suppressed ‘Royal Gala’ apples were firmer than wild-type after ripening. However, in this experiment, *PG1* expression was weak during the process of ‘Ruanrou Shanlihong 3 Hao’ fruit development, but it was higher in the hard-fleshed hawthorn at the late stage of fruit development. This result is consistent with the TEM observations of cell walls at the late developmental stage. Another glycosidase, α*-ARF*, has been well documented for its involvement in cell wall modifications during fruit ripening. α*-ARF* has been proposed to release arabinosyl residues from the pectic fraction, and its functions have been widely discussed in melon (Nishiyama et al., 2007), apple (Ireland et al., 2014), and pear (Mwaniki et al., 2007). However, the patterns of α*-ARF* expression were similar to those of *PG1* in this study, indicating that this enzyme is not part of the softening process. The level of *EG17* expression exhibited a decreasing tendency in both ‘Qiu JinXing’ and ‘Ruanrou Shanlihong 3 Hao’ fruits, and the levels were nearly the same in the two cultivars at the late developmental stages. The level of *GDP-man* expression showed a decreasing tendency with fruit developmental stage in ‘Qiu JinXing’ fruits. At the middle developmental stage, the levels in the two cultivars were nearly the same. At the late developmental stage, the level of *GDP-man* expression in ‘Qiu JinXing’ fruits rapidly decreased and was lower than that in ‘Ruanrou Shanlihong 3 Hao’ fruits. *XTH* expression in ‘Qiu JinXing’ fruits was higher than that in ‘Ruanrou Shanlihong 3 Hao’ fruits in early developmental stageI; however, the level of *XTH* expression in ‘Ruanrou Shanlihong 3 Hao’ fruits was higher than that in ‘Qiu JinXing’ fruits in the other developmental stages. Takeda et al. (2015) found that *Gns* plays a significant role in the degradation and modification of β-1, 3-glucan in fungal cell walls, and this is consistent with our results. The expression level of *Gns* in our study was high in soft-fleshed hawthorn fruits but weak in hard-flesh hawthorns at the late developmental stage. Overall, *PG1*, α*-ARF*, and *EG17* might not participate in textural changes in hawthorn fruits, whereas *GDP-man, XTH*, and *Gns* might be important for changing fruit texture in hawthorn fruits. Thus, the mechanisms by which hydrolytic enzymes affect fruit softening are distinct and complex for different types of fruit.

## Conclusion

We hypothesize that different types of hydrolytic enzymes in cells interacted to degrade the cell wall, which results in different degrees of cell wall degradation and result in fruit softening, which can be assessed by TEM and RNA-Seq. These results of this study suggest that soft and hard flesh textures in *C. pinnatifida* fruits are resulted from different degrees of cellulose/hemicelluloses degradation. These results could provide fundamental insights regarding the mechanisms responsible for different hawthorn fruit textures and lay a solid foundation for further in-depth research.

## Author Contributions

JX and WD conceived this project and designed the work. JX, YZ, and LZ performed the research. JX, XZ analyzed data, JX, WD, and YH wrote the manuscript.

## Conflict of Interest Statement

The authors declare that the research was conducted in the absence of any commercial or financial relationships that could be construed as a potential conflict of interest.

## Supplementary material

FIGURE S1 **Sequence of the amplification products for real-time PCR**.

FIGURE S2 **Nr function classification of assembled unigenes.** The species distribution is shown relative to the total number of homologous sequences.

FIGURE S3 **KOG function classification of the assembled unigenes.** Overall, 12,143 unigenes were grouped into 25 COG classifications.

FIGURE S4 **Differential gene expression profiles for different comparisons. (A)** for RRSIVand QJXIV and **(B)** for RRSV and QJXV. Scatter plot of DEGs (FDR < 0.01 and FC ≥ 2) illustrating the full set of genes sampled. Red points: down-regulated genes; blue points: up-regulated; green points: genes that are not DEGs.

FIGURE S5 **(A)** Enriched top GO directed acyclic graph (DAG) of significant DEGs (molecular function) between RRSIV and QJXIV. **(B)** Enriched top GO directed acyclic graph (DAG) of significant DEGs (molecular function) between RRSVand QJXV.

TABLE S1 **DEGs related to the cell wall in different textures of ‘Ruanrou Shanlihong 3 Hao’ and ‘Qiu JinXing’ fruits at two developmental stages**.

TABLE S2 **Primers for real-time PCR**.
