# Supplementary material for: Transcriptome Analysis and Ultrastructure Observation Reveal that Hawthorn Fruit Softening Is due to Cellulose/Hemicellulose Degradation
Source: Front Plant Sci. 2016 Oct 14;7:1524. doi: 10.3389/fpls.2016.01524 (PMC5063854; doi:10.3389/fpls.2016.01524)
Supplement: FIGURE S1 — Sequence of the amplification products for real-time PCR. [file Image_1.PDF]

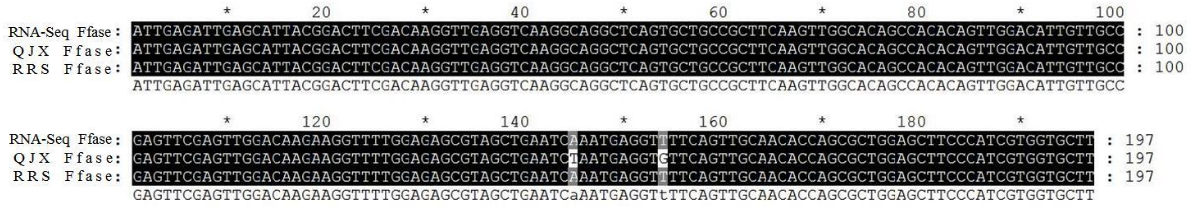

Alignments at the cDNA level, RNA-Seq as a reference

Identity for *Ffase*: QJX: 195/197*100%=99.0%; RRS: 197/197*100%=100%

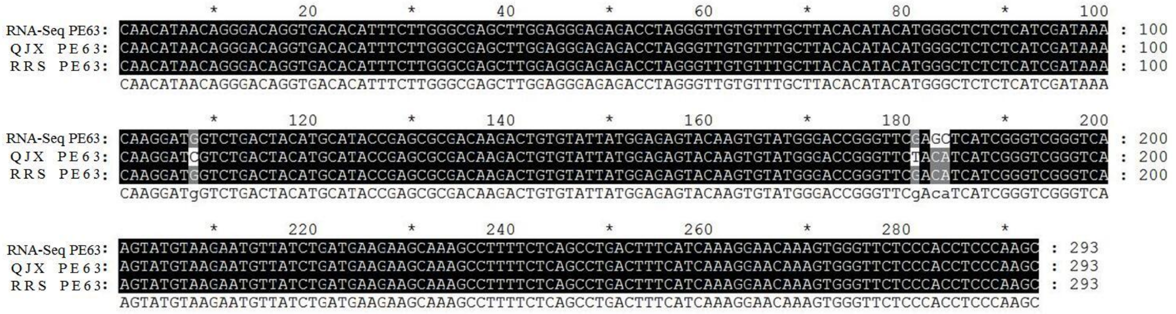

Alignments at the cDNA level, RNA-Seq as a reference

Identity for *PE63*: QJX: 289/293*100%=98.6%; RRS: 291/293*100%=99.3%

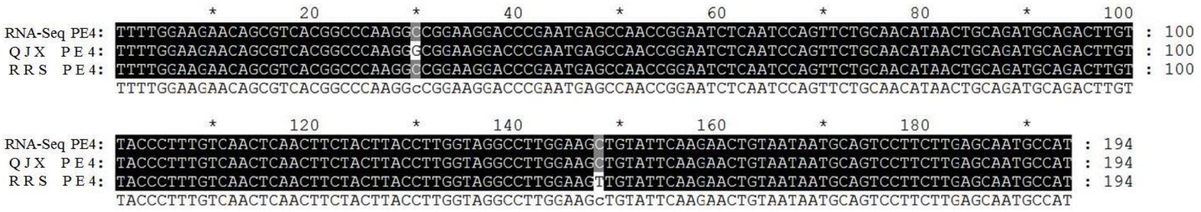

Alignments at the cDNA level, RNA-Seq as a reference

Identity for *PE4*: QJX: 193/194*100%=99.5%; RRS: 193/194*100%=99.5%

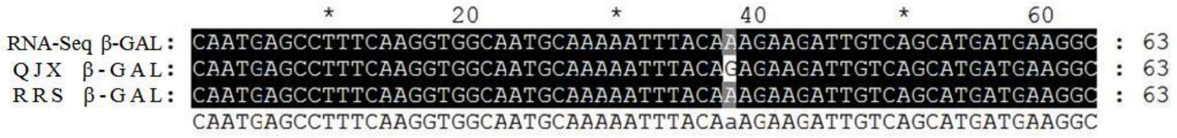

Alignments at the cDNA level, RNA-Seq as a reference

Identity for *β-GAL*: QJX: 62/63*100%=98.4%; RRS: 63/63*100%=100%

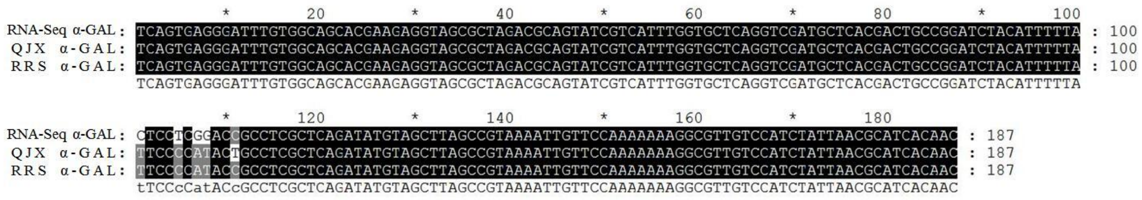

Alignments at the cDNA level, RNA-Seq as a reference

Identity for *α-GAL*: QJX: 182/187*100%=97.3%; RRS: 183/187*100%=97.9%

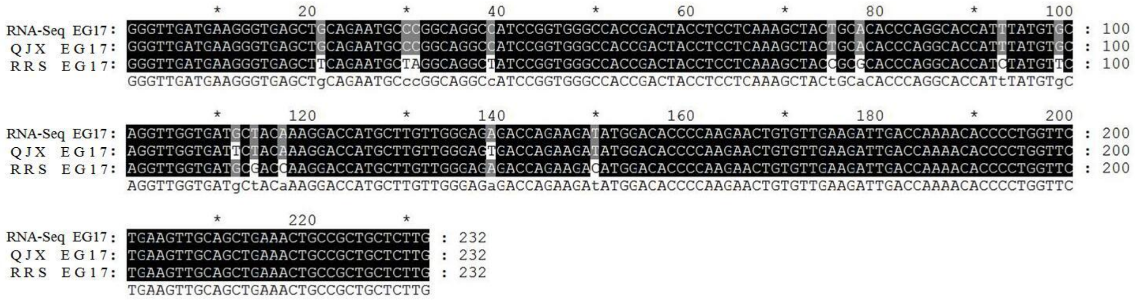

Alignments at the cDNA level, RNA-Seq as a reference

Identity for *EG17*: QJX: 230/232*100%=99.1%; RRS: 221/232*100%=95.3%

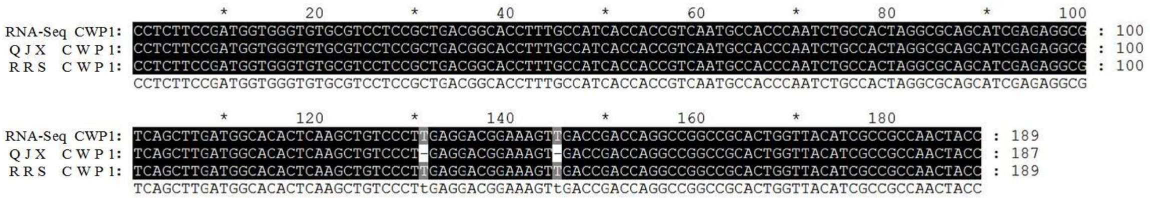

Alignments at the cDNA level, RNA-Seq as a reference

Identity for CPW1: QJX: 187/189*100%=99.0%; RRS: 189/189*100%=100%

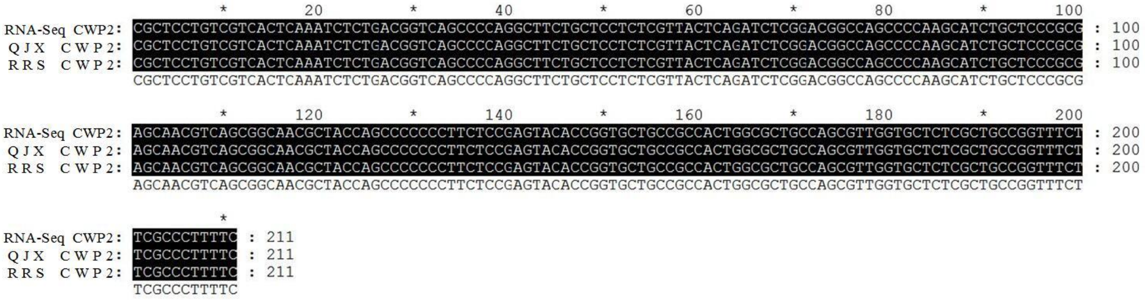

Alignments at the cDNA level, RNA-Seq as a reference

Identity for CPW2: QJX: 211/211*100%=100%; RRS: 211/211*100%=100%

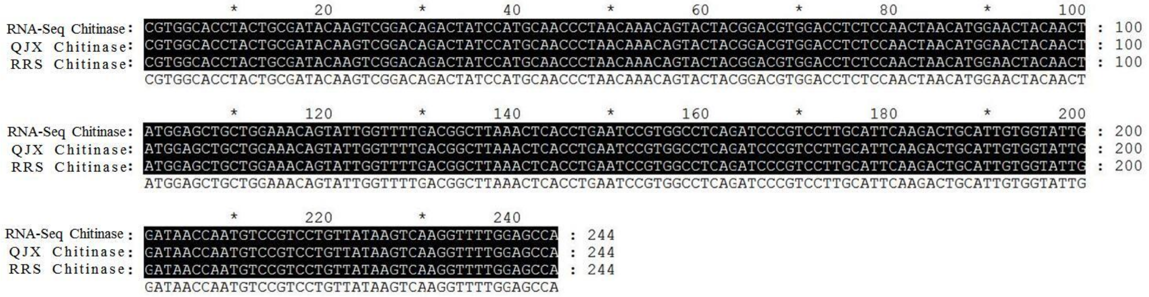

Alignments at the cDNA level, RNA-Seq as a reference

Identity for *Chitinase*: QJX: 244/244*100%=100%; RRS: 244/244*100%=100%

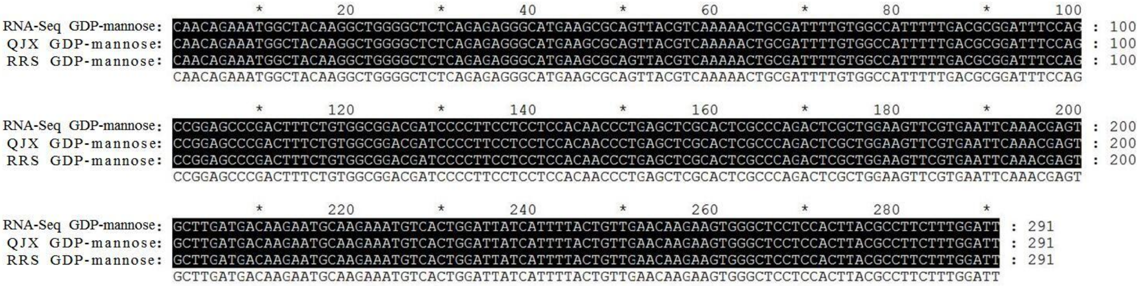

Alignments at the cDNA level, RNA-Seq as a reference

Identity for *GDP-mannose*: QJX: 291/291*100%=100%; RRS: 291/291*100%=100%

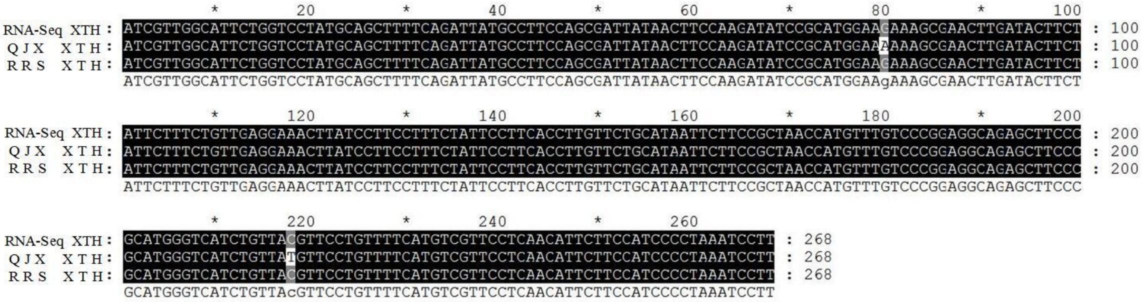

Alignments at the cDNA level, RNA-Seq as a reference

Identity for *XTH*: QJX: 266/268*100%=99.3%; RRS: 268/268*100%=100%

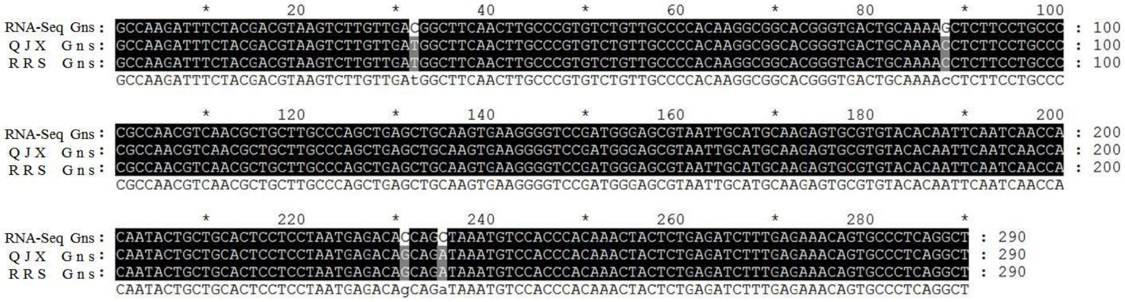

Alignments at the cDNA level, RNA-Seq as a reference

Identity for *Gns*: QJX: 286/290*100%=98.6%; RRS: 286/290*100%=98.6%

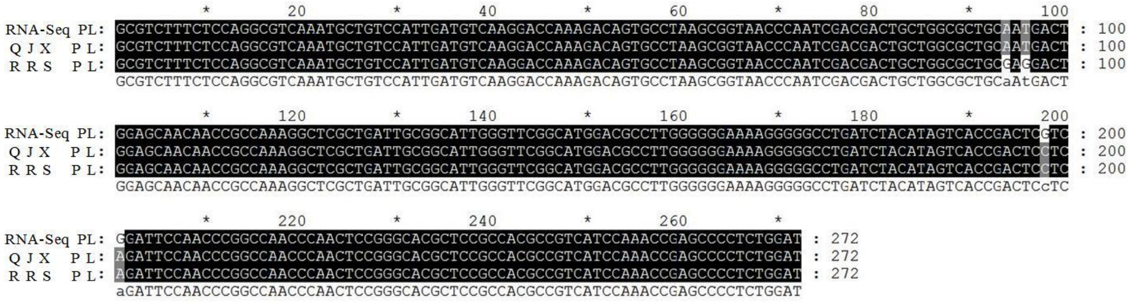

Alignments at the cDNA level, RNA-Seq as a reference

Identity for *PL*: QJX: 270/272*100%=99.3%; RRS: 268/272*100%=98.5%

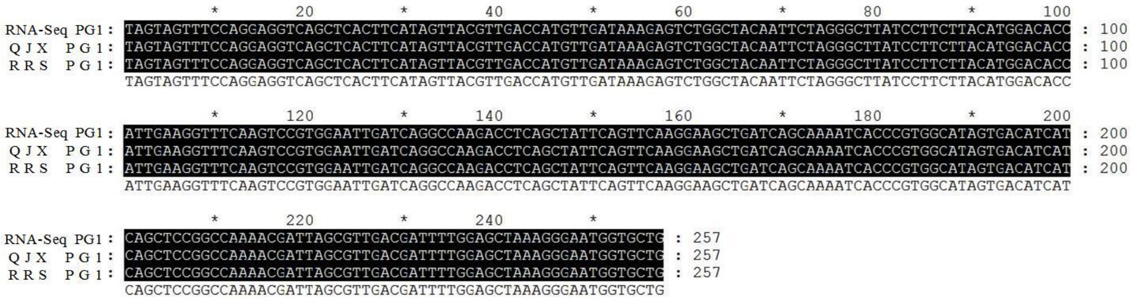

Alignments at the cDNA level, RNA-Seq as a reference

Identity for *PG1*: QJX: 257/257*100%=100%; RRS: 257/257*100%=100%
