# Supplementary figures and images for: Transcriptome Analysis and Ultrastructure Observation Reveal that Hawthorn Fruit Softening Is due to Cellulose/Hemicellulose Degradation
Source: Front Plant Sci. 2016 Oct 14;7:1524. doi: 10.3389/fpls.2016.01524 (PMC5063854; doi:10.3389/fpls.2016.01524)

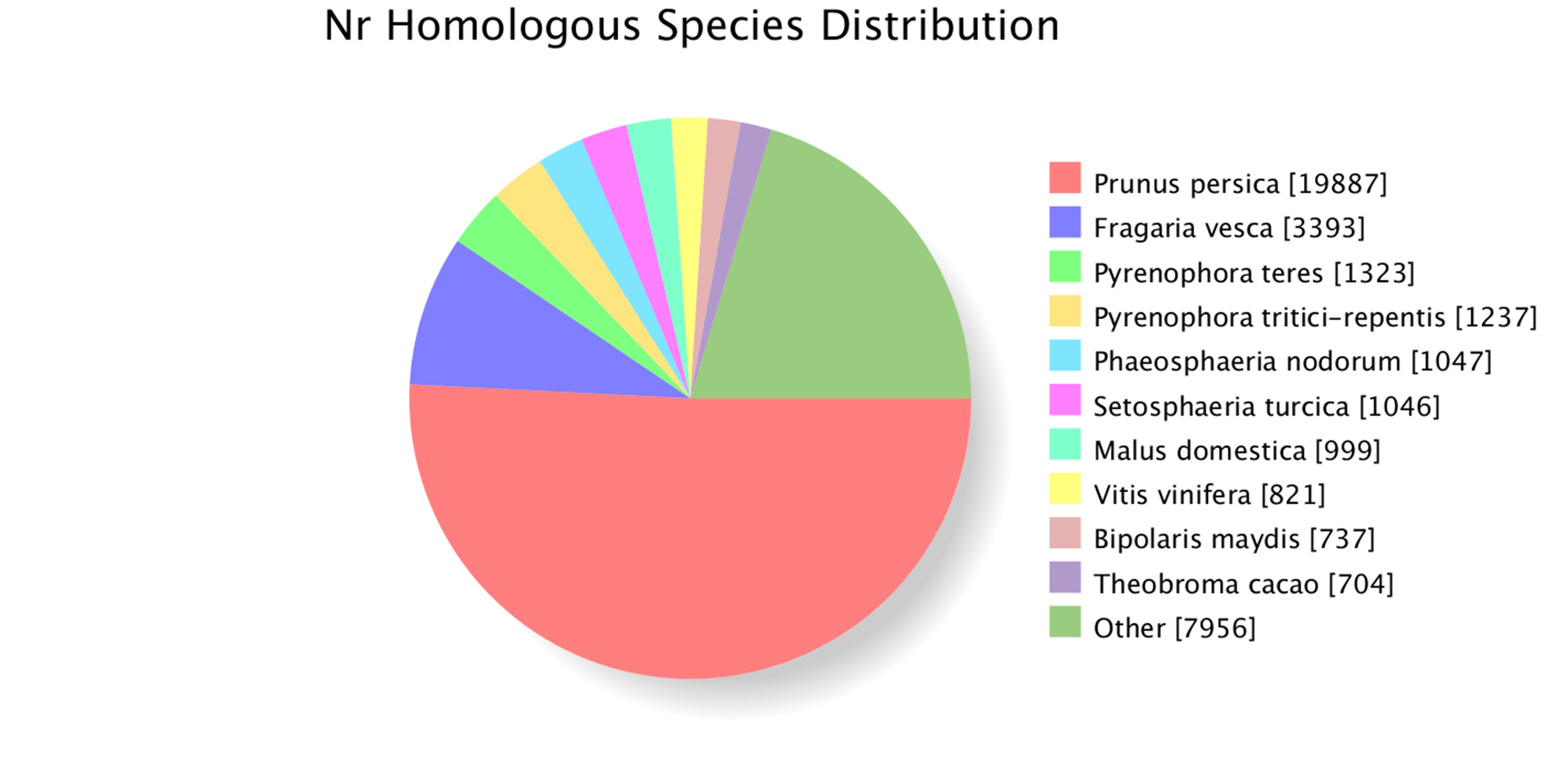

Supplement: FIGURE S2 — Nr function classification of assembled unigenes. The species distribution is shown relative to the total number of homologous sequences. [file Image_2.JPEG]

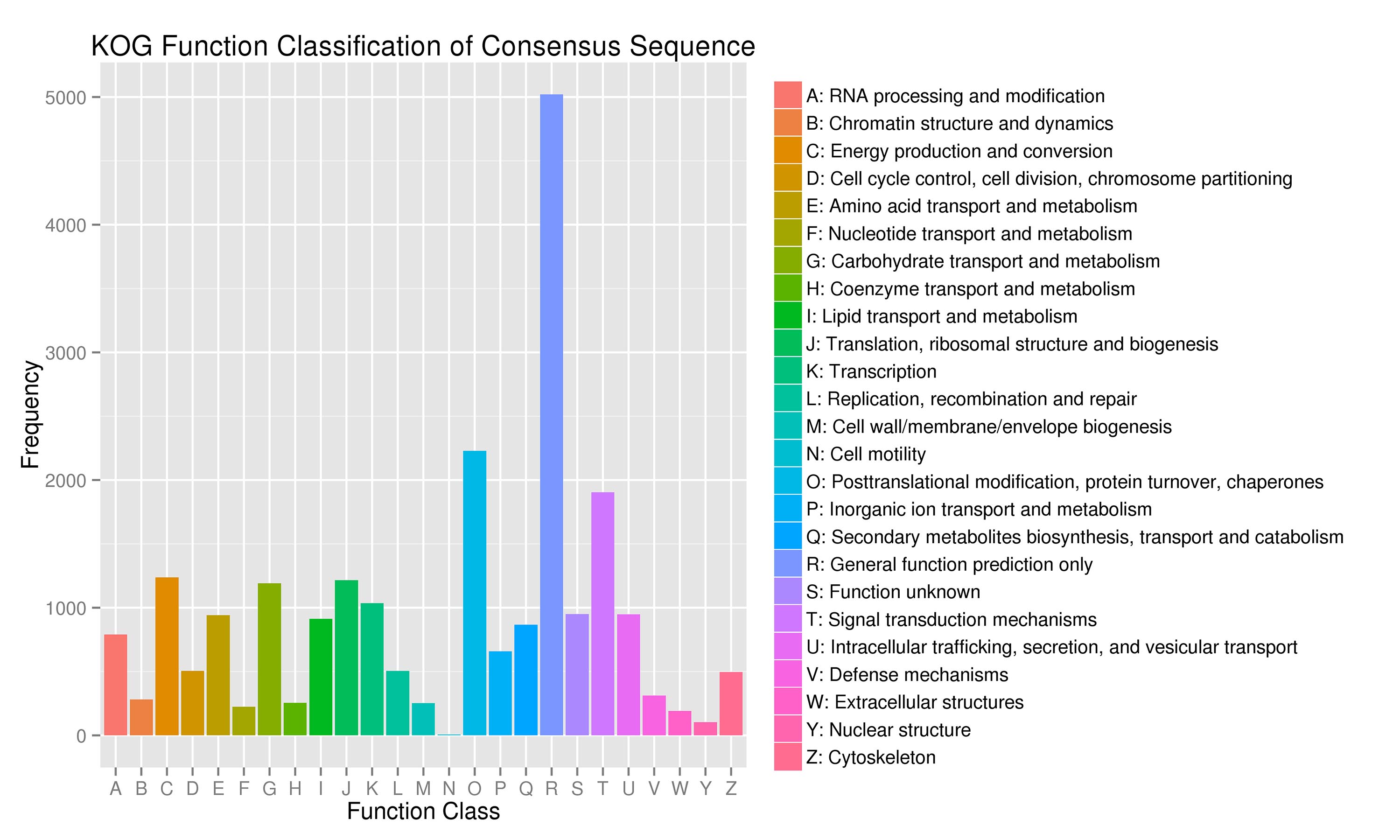

Supplement: FIGURE S3 — KOG function classification of the assembled unigenes. Overall, 12,143 unigenes were grouped into 25 COG classifications. [file Image_3.JPEG]

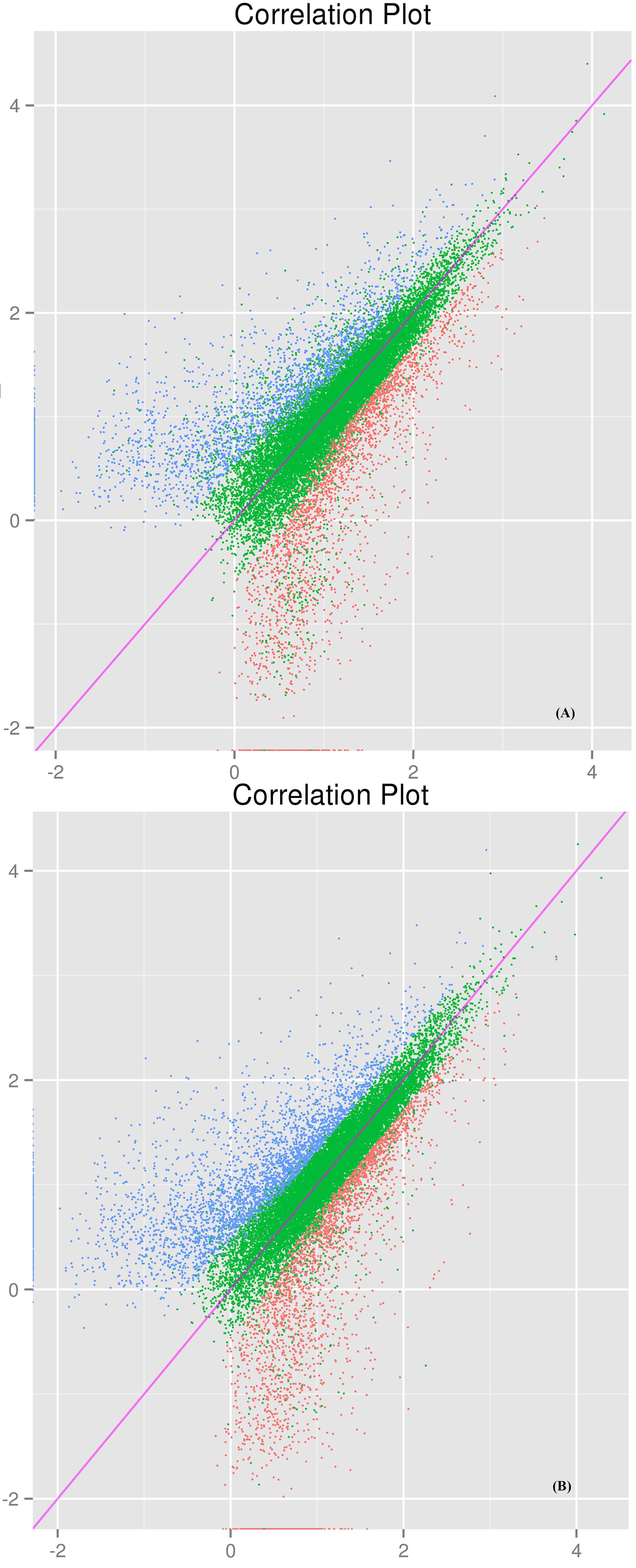

Supplement: FIGURE S4 — Differential gene expression profiles for different comparisons. (A) for RRSIVand QJXIV and (B) for RRSV and QJXV. Scatter plot of DEGs (FDR < 0.01 and FC ≥ 2) illustrating the full set of genes sampled. Red points: down-regulated genes; blue points: up-regulated; green points: genes that are not DEGs. [file Image_4.JPEG]

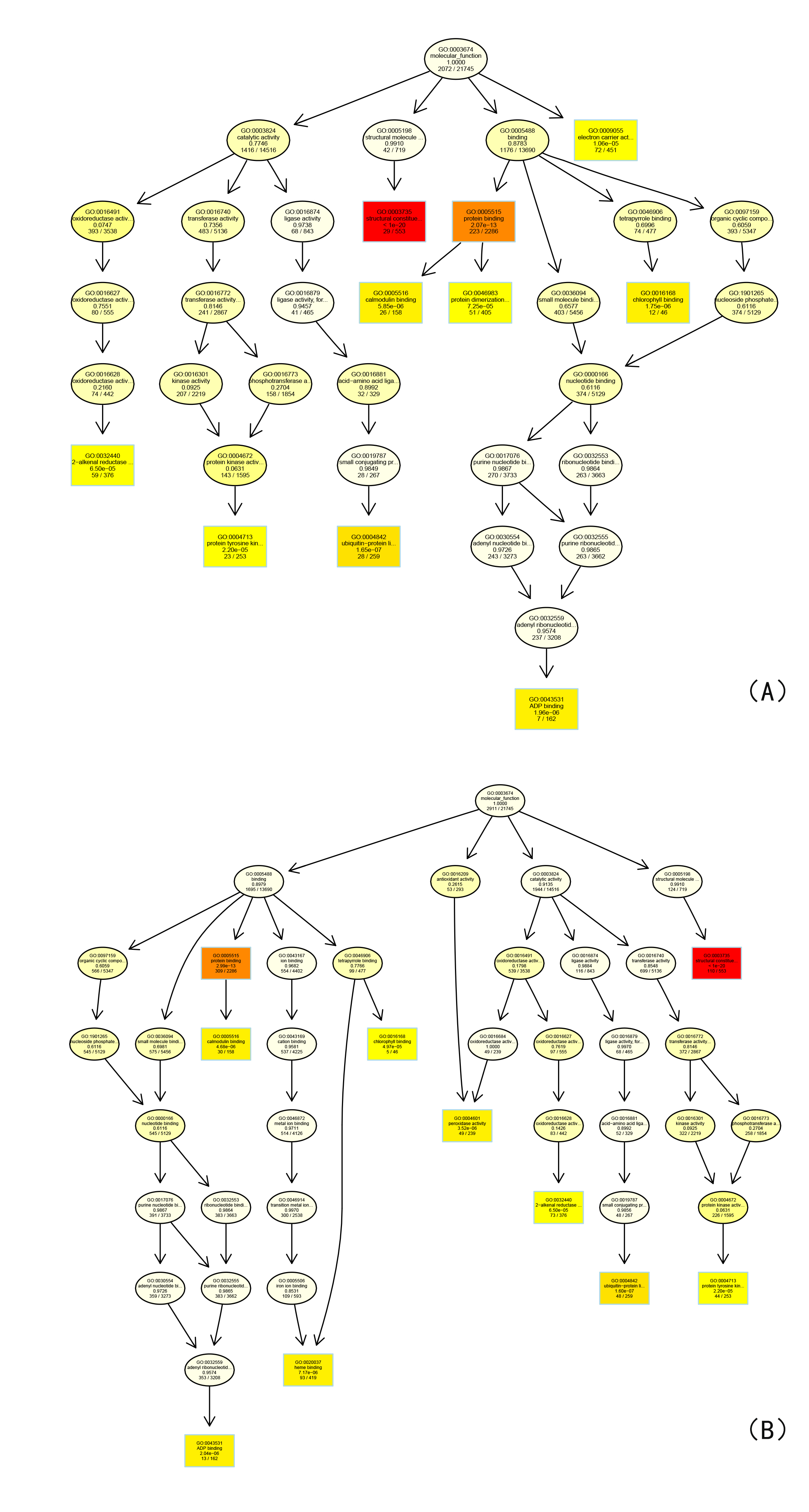

Supplement: FIGURE S5 — (A) Enriched top GO directed acyclic graph (DAG) of significant DEGs (molecular function) between RRSIV and QJXIV. (B) Enriched top GO directed acyclic graph (DAG) of significant DEGs (molecular function) between RRSVand QJXV. [file Image_5.JPEG]
